# Comprehensive Analysis of RELL2 as a Potential Biomarker Associated with Tumor Immune Infiltrating Cells in a Pan-Cancer Analysis

**DOI:** 10.1155/2022/5009512

**Published:** 2022-05-18

**Authors:** Kadeerjiang Musha, Xinqi Ge, Nuraliya Ablikim, Bing Lu, Chen Chen, Jianfei Huang

**Affiliations:** ^1^Department of Cardiothoracic Surgery, People's Hospital of Kizilsu Kirgiz Autonomous Prefecture, Kizilsu Kirgiz, 845350 Xinjiang, China; ^2^Department of Clinical Biobank & Institute of Oncology, Affiliated Hospital of Nantong University, Nantong, 226001 Jiangsu, China; ^3^Translational Medicine Center, People's Hospital of Kizilsu Kirgiz Autonomous Prefecture, Kizilsu Kirgiz, 845350 Xinjiang, China; ^4^Department of Oncology, Jiangsu Cancer Hospital & Jiangsu Institute of Cancer Research & The Affiliated Cancer Hospital of Nanjing Medical University, Nanjing 210000, China; ^5^The Comprehensive Cancer Centre of Nanjing Drum Tower Hospital, The Affiliated Hospital of Nanjing University Medical School & Clinical Cancer Institute of Nanjing University, 210008, China

## Abstract

**Background:**

Receptor expressed in lymphoid tissues-like 2 (RELL2), which is a member of RELT family, is closely associated with the plasma membrane and acts as a modulator for RELT signaling. Overexpression of RELL2 induces the activation of MAPK14/p38 cascade and apoptosis. However, whether RELL2 contributes to cancers remains unclear. Here, we examined its role in cancer patient prognosis and various tumors.

**Methods:**

We used several bioinformatics methods, specifically gene set enrichment analysis (GSEA), ScanNeo, and ESTIMATE, to analyze the CCLE dataset, GTEx dataset, and TCGA dataset. We investigated the possible association of RELL2 with the microsatellite instability (MSI) of various tumors, tumor mutational burden (TMB), immune checkpoint, immune neoantigens, immune microenvironment, and patient prognosis.

**Result:**

RELL2 is highly expressed in cancer compared with normal tissues. RELL2 expression is linked with worse progression-free interval and overall survival in numerous cancers. In most cancers, high RELL2 expression was related to a poor prognosis. RELL2 expression was significantly associated with the tumor microenvironment, MSI, and TMB. RELL2 expression is strongly associated with phenotypes that are of major clinical significance, particularly those associated with immune neoantigens and the expression profiles of immune checkpoint genes in pan-cancer. RELL2 expression strongly linked with the expressions of methyltransferases and DNA repair genes. It also significantly correlated with multiple signaling pathways through gene set enrichment analysis.

**Conclusion:**

RELL2 may be a prognostic biomarker in pan-cancer and may have an important function in tumorigenesis and progression.

## 1. Introduction

Receptor expressed in lymphoid tissues-like 2 (RELL2), a homologue of RELT, has been confirmed as tumor necrosis factor receptor (TNFR). Overexpression of RELL2 leads to cell death in human epithelial cells by activating an apoptotic pathway [1, 2]. Moreover, RELL2 suppresses the metastatic ability of MDA-MB-231 and 4T1 cells and is a target of miR-18a [3]. RELL2 expression has been detected in breast, brain, placenta, thymus, spleen, and testis cancer. Several reports demonstrated that RELL2 exhibits antitumor activity; for example, RELL2 reduces breast cancer cell invasion and migration and inhibits the tumorigenesis of esophageal cancer cells [3, 4].

Recent reports confirmed that tumor necrosis factor activates innate immunity and mediates the transition to adaptive immunity [5], making mitochondria more easily to be collapsed by mitochondrial membrane potential [6] and inducing an inflammatory cascade [7]. TNFR is involved in inducing apoptosis [8]. Apoptosis can be used in disorders involving in overproliferation so that it can control cell proliferation and keep cell numbers at a constant number [9]. Apoptosis is initiated by specific receptors of the tumor necrosis factor superfamily [9]. To induce tumor cell apoptosis without affecting noncancerous cells, an important goal for cancer therapeutics, tumor necrosis factor–related apoptosis-inducing ligand (TRAIL) has been explored [10, 11].

RELL2 plays a crucial role in the tumor immune system and induces apoptosis. Nevertheless, its role in activating apoptosis has yet to be evaluated in the context of tumor immunity and metabolism. Pan-cancer analysis has generated important findings. This study unveils the mechanism underlying RELL2 in pan-cancer according to the data obtained from various datasets.

In recent years, the application of immunotherapy as a modern cancer therapy has been increasingly expanded in clinical applications [12, 13]. Anticancer immunotherapy encounters its own opportunity, and several reports identified biomarkers of immune checkpoint inhibitors such as checkpoint-ligand expression, DNA repair deficiency, and mutational burden [14]. Immunotherapy has rapidly emerged as a cornerstone in the treatment methods of many cancers, but the response to immunotherapy is not very optimistic and only some patients obtain a durable response to immunotherapy [15, 16]. Thus, better understanding of mechanisms of immunotherapy towards cancer is necessary [17].

In this study, we performed an exhaustive pan-cancer analysis to examine the possible role of RELL2 in cancers and the associations of its expression with prognosis. Our results demonstrated a link between RELL2 expression and immune neoantigens, immune checkpoint genes (ICGs), microsatellite instability (MSI), tumor mutation burden (TMB), and the immune microenvironment. The associations between RELL2 expression and four methyltransferases (DNMT3B, DNMT3A, DNMT2, and DNMT1) were assessed.

## 2. Materials and Methods

### 2.1. Data Collection

We used the Genotype-Tissue Expression (GTEx) (https://gtexportal.org/) and The Cancer Genome Atlas (TCGA) (https://portal.gdc.cancer.gov/) databases to obtain the gene expression profiles and clinical data. We also used The Cancer Cell Line Encyclopedia (CCLE) database (https://portals.broadinstitute.org/) to download 21 types of tumor cell lines. From TCGA database, we obtained 20 tumor samples and we obtained data of 31 tumor organizations from the GTEx database. We further collected data on 35 types of human cancers from the TIMER database (https://cistrome.shinyapps.io/timer/).

### 2.2. RELL2 Gene Expression Analysis

Using the edgeR software, we analyzed RELL2 expression levels in normal tissues and adjacent tumor tissues based on the TCGA database. To expand the cancer types and sample sizes, we combined expression data from the TCGA and GTEx databases. RELL2 expression levels between various tissues from the GTEx database and CCLE database were analyzed with Kruskal–Wallis test. The violin plots were visualized by R package ggplot.

### 2.3. Differences between RELL2 Expression and Survival in Cancers

Using 33 cancer types from the TCGA database, we studied the links between RELL2 expression and overall survival (OS) through the univariate Cox model. Statistical significance was indicated as *P* < 0.05. We used Kaplan–Meier (KM) method to evaluate PFI (progression-free interval), DFI (disease-free interval), DSS (disease-specific survival), and OS (overall survival) in patients with high and low expression levels of RELL2 across various cancer types.

### 2.4. Correlations of RELL2 Expression with the Immune Microenvironment

We examined the relation between RELL2 expression levels and six different immune infiltrating levels (macrophages, dendritic cells (DCs), neutrophils, B cells, CD8+ T cells, and CD4+ T cells) using the TIMER database to obtain the score data of the latter, including the gene expression profiles of 32 cancer types [18]. The estimate R package was used to explore the abundance of immune and stromal components. *P* < 0.05 and *R* > 0.20 indicated a significant and positive association, respectively, with the expression level of RELL2.

### 2.5. Relationships among RELL2 Expression Level and ICGs and Immune Neoantigens

Mutated genes of tumor cells encode new antigens called neoantigens. Such genes are primarily created through other abnormal proteins, gene fusions, deletions, and point mutations, all of which are distinct from proteins expressed by normal cells [19]. Several studies have explored the use of neoantigen vaccines to improve the response of the immune system to cancer cells [20]. We examined the correlation of RELL2 expression level with the number of antigens that were counted separately [19]. We extracted more than 40 common internal control genes (ICGs) to investigate the correlation of RELL2 with ICGs. *P* < 0.05 and *R* > 0.20 indicated a significant and positive association, respectively.

### 2.6. Connections between RELL2 Expression and TMB and MSI

TMB refers to the quantity of nonsynonymous mutations per coding region of a tumor genome identified in whole-exome sequencing through a reported algorithm, including the somatic variants per megabase (MB) of the genome [21, 22]. MSI occurs when some cells perform one or two alleles along with different numbers of repeats and has been identified to be associated with clinicopathological characteristics of cancer patients [23, 24]. The relationships between RELL2 expression with TMB and MSI were analyzed with Spearman's rank correlation coefficient.

### 2.7. Associations between RELL2 Expression and DNA Mismatch Repair (MMR) Genes and Methyltransferases

EPCAM, PMS2, MSH6, MSH2, and MLH1 are DNA MMR genes used to forecast immune checkpoint inhibitors to strengthen the immune response to new anticancer therapies. The inactivation of DNA MMR genes played a role in the pathology of specific sporadic and hereditary cancers [25]. We downloaded data from the TCGA database to explore the correlation of RELL2 expression with MMRs.

DNA methylation is mediated by DNA methyltransferases (DNMTs) and can be influenced by the environment [26–28]. We analyzed the association of RELL2 expression with four methyltransferases (DNMT3B, DNMT3A, DNMT2, and DNMT1). *P* < 0.05 and *R* > 0.20 indicated a significant and positive association, respectively.

### 2.8. Gene Set Enrichment Analysis (GSEA)

We used GSEA to distinguish a group of genes statistically enriched for a particular observable variable and determine biological processes associated with groups of differentially expressed genes [29]. Using the Molecular Signature Database linked with various expressions under different conditions from the Broad Institute (http://www.broadinstitute.org/GSEA/msigdb/index.jsp), we applied the GSEA to analyze the distributions of the supervised gene sets [30]. We used Kyoto Encyclopedia of Genes and Genomes (KEGG) Automatic Annotation Server to perform KEGG pathway analysis. KEGG incorporates a variety of databases on genomes, drugs, diseases, chemical substances, and biological pathways [31]. We then employed signature collections from Molecular Signature Database, specifically “hallmark” and “KEGG,” to carry out the analysis.

## 3. Results

### 3.1. Pan-Cancer Analysis of RELL2 Expression Levels

**Figure 1:**
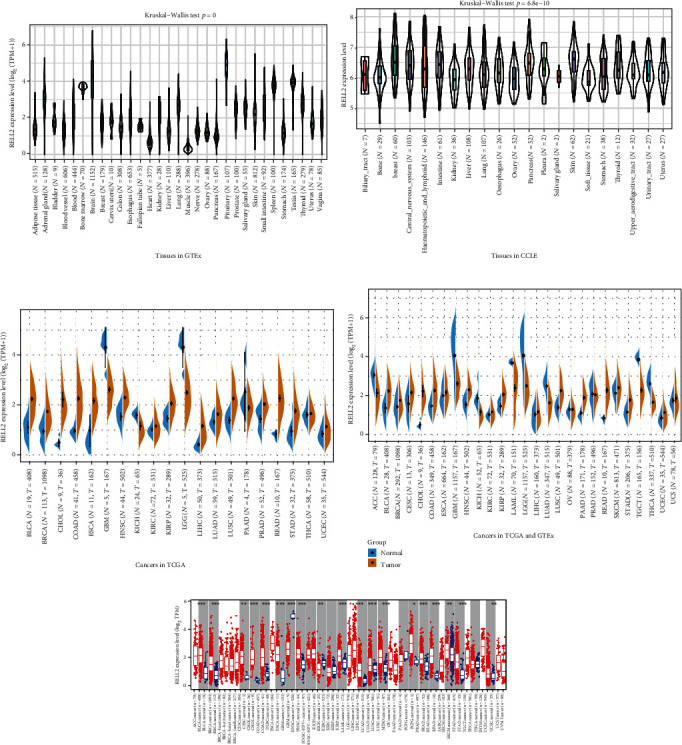
RELL2 expression level across various cancer types. (a) RELL2 expression in 31 tissues in the GTEx database. (b) RELL2 expression in 21 tumor cell lines/tissues in the CCLE database. (c) RELL2 expression level in normal and tumor tissues in the TCGA database. (d) RELL2 expression in normal and tumor tissues in GTEx and TCGA databases. (e) RELL2 expression in normal and tumor tissues in the TIMER database.

We first investigated the expression level of RELL2 among different tissues based on the GTEx (Genotype-Tissue Expression) dataset and found that RELL2 (Receptor expressed in lymphoid tissues-like 2) showed varying expression levels in 31 tissues (Figure 1(a)). We then explored RELL2 expression level in tumor cell lines using data from the CCLE (The Cancer Cell Line Encyclopedia) database (Figure 1(b)). We investigated RELL2 expression level in pan-cancer data from the TCGA (The Cancer Genome Atlas) dataset and found that RELL2 was expressed higher in 16 out of 20 cancers. The highest RELL2 expression was detected in rectum adenocarcinoma (READ) and neck squamous cell carcinoma (HNSC) (Figure 1(c)).

Given that TCGA database contains few normal tissues, we merged it with the GTEx database to examine RELL2 expression level in various tumor and adjacent normal tissues. RELL2 was expressed higher in 18 out of 27 or in the majority of cancer types (Figure 1(d)). Moreover, we used the TIMER (Tumor Immune Estimation Resource) database to compare the RELL2 expression level of tumor tissues with that of normal tissue (Figure 1(e)).

### 3.2. Correlations between RELL2 Expression and Patient Prognostic Outcomes

**Figure 2:**
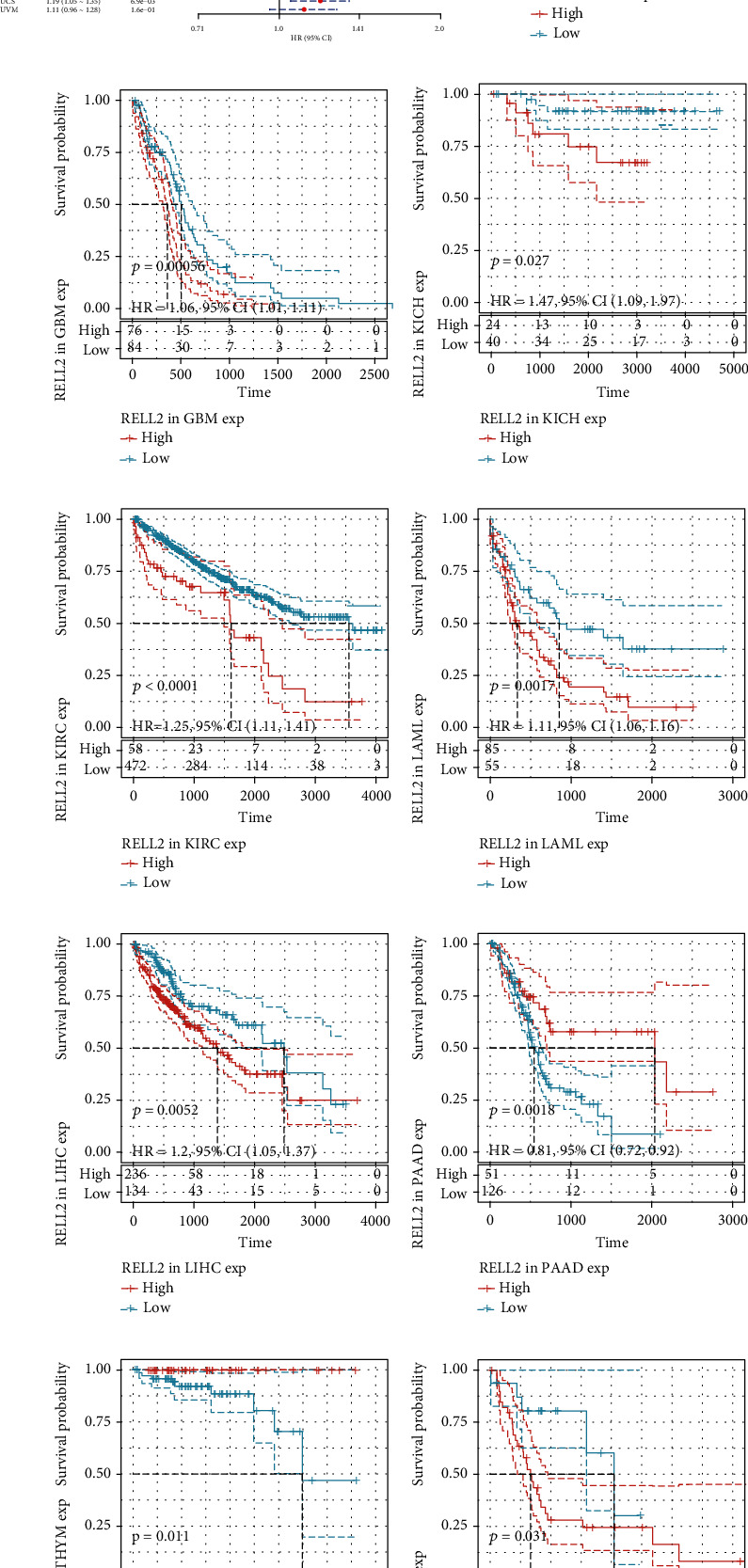
Forest plot and Kaplan-Meier OS curves of RELL2 expression in TCGA database. (a) The relationships between RELL2 expression and OS (overall survival) in 33 kinds of cancers. (b–j) Kaplan-Meier analysis of OS on the basis of RELL2 expression and 33 types of cancers.

**Figure 3:**
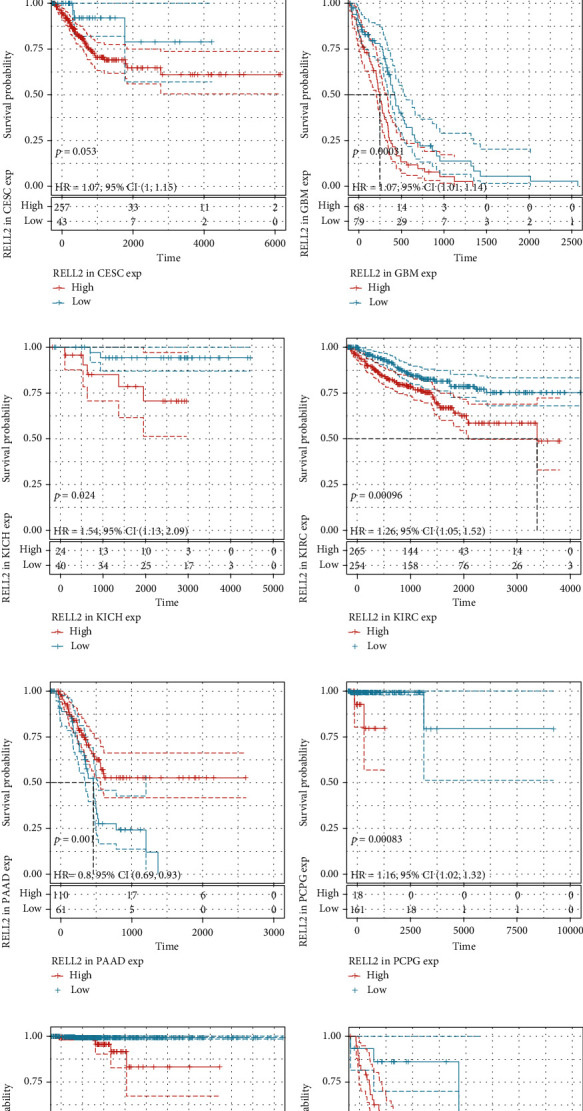
Forest plot and Kaplan-Meier DSS curves of RELL2 expression in TCGA database. (a) The relationships between RELL2 expression and 33 kinds of cancers associated with DSS (disease-specific survival). (b–j) Kaplan–Meier analysis of disease-specific survival outcomes on the basis of RELL2 expression in 33 types of cancers.

**Figure 4:**
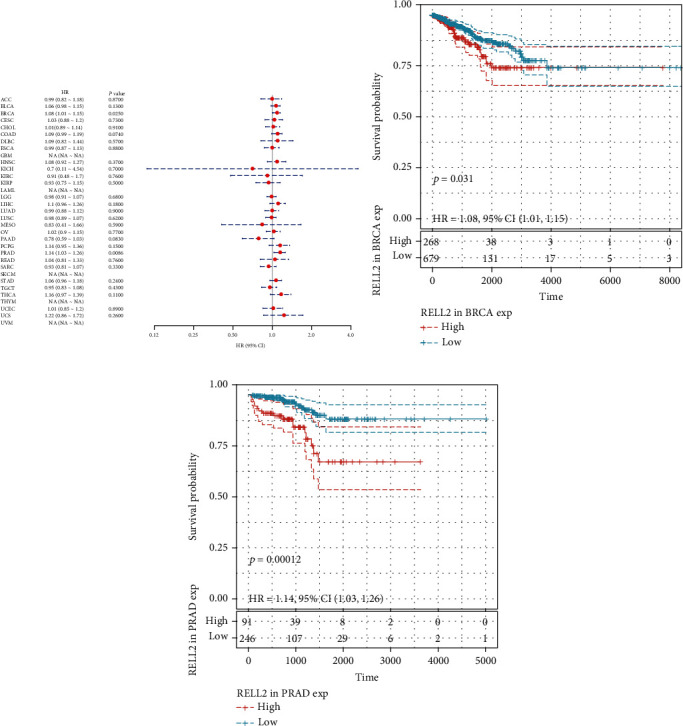
Forest plot and Kaplan-Meier DFI curves of RELL2 expression in TCGA database. (a) The relationships between RELL2 expression and 33 kinds of cancers associated with DFI (disease-free interval). (b, c) Kaplan–Meier analysis results on disease-free interval outcomes on the basis of RELL2 expression in 33 types of cancers.

**Figure 5:**
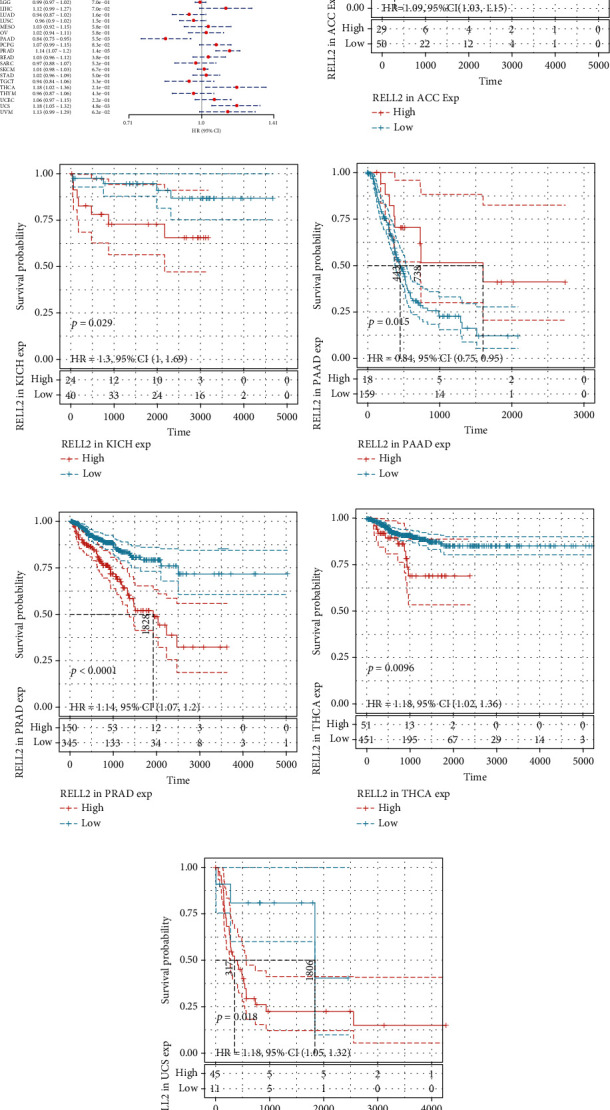
Forest plot and Kaplan-Meier PFI curves of RELL2 expression in TCGA database. (a) The relationships between RELL2 expression and 33 kinds of cancers associated with PFI (progression-free interval). (b–g) The most significant Kaplan-Meier analysis results regarding to the progression-free interval outcomes between RELL2 expression and 33 types of cancers.

To examine the influence of RELL2 expression on patient prognosis, we used the TCGA database and performed univariate Cox analysis to explore the relationship of RELL2 expression level with PFI, DFI, DSS, and OS. RELL2 significantly influenced OS in ACC, GBM, KICH, KIRC, LAML, LIHC, PAAD, and THYM (Figure 2(a)). The most significant OS outcomes associated with the expression of RELL2 are shown in Figure 2(b). A shorter OS was associated with RELL2 expression levels in ACC, GBM, KICH, KIRC, LAML, LIHC, and UCS while RELL2 expression was associated with a better OS outcome in PAAD and THYM. Forest plots revealed that RELL2 affected the DSS of ACC, GBM, KICH, KIRC, PAAD, PCPG, THCA, and UCS (Figure 3(a)). High expression level of RELL2 predicted a poor survival outcome in ACC, CESC, GBM, KICH, KIRC, PCPG, THCA, and UCS and indicated a good survival in PAAD (*P* = 0.001) (Figure 3(b)). Forest plots revealed that RELL2 influenced DFI in BRCA and PRAD (Figure 4(a)). And the most significant survival outcomes revealed that high expression levels of RELL2 predicted a poor survival outcome in BRCA and PRAD (Figure 4(b)). Forest plots revealed that RELL2 influenced PFI of ACC, PAAD, PRAD, THCA, and UCS (Figure 5(a)). And the most significant survival outcomes revealed that high expression level of RELL2 predicted a poor survival outcome in ACC, KICH, PRAD, THCA, and UCS and predicted a good survival outcome in PAAD (Figure 5(b)).

### 3.3. RELL2 Expression between Different Clinical Characteristics

**Figure 6:**
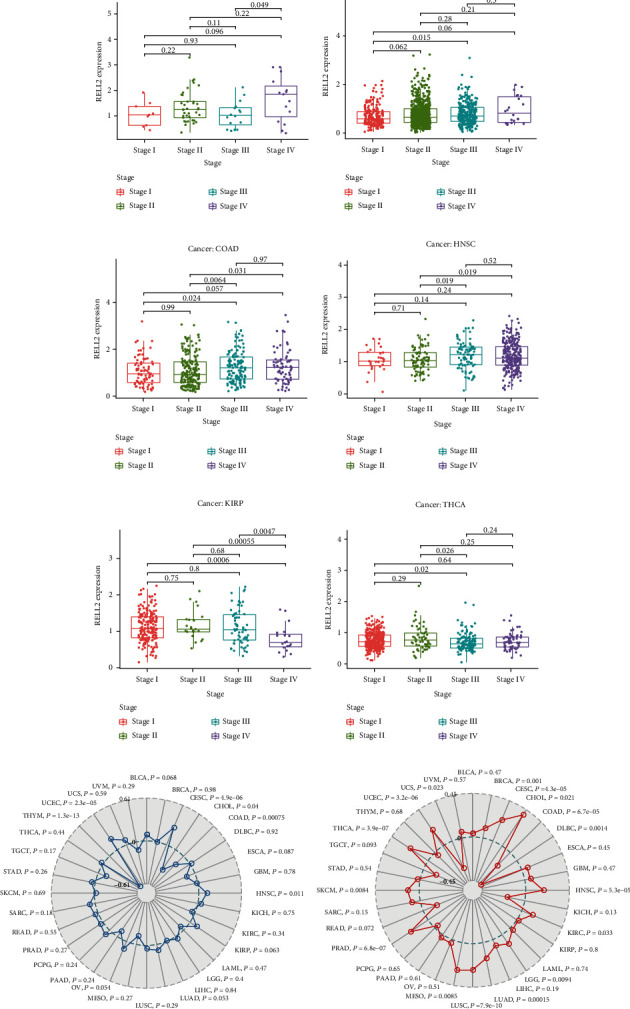
The relationships between RELL2 expression and different stages among 32 cancers and TMB, MSI in theTCGA database. (a–f) Distinct pathological stages of the most significant RELL2 expression levels across various cancer types. (g) Correlations of RELL2 expression levels with TMB. (h) Correlations of RELL2 expression levels and MSI.

We next examined the association of RELL2 expression with pathological stage in pan-cancers, MSI, and TMB status from the TCGA database. A significant relationship between RELL2 expression and tumor stage was found in THCA, KIRP, HNSC, COAD, BRCA, and ACC (Figures 6(a)–6(f)). RELL2 expression level was significantly correlated with TMB in UCEC, THYM, COAD, and CESC (Figure 6(g)) and significantly correlated with MSI in 16 cancer types, including UCEC, THCA, PRAD, LUSC, LUAD, HNSC, COAD, and BRCA (Figure 6(h)). Collectively, these results identify RELL2 as a potential prognostic biomarker for various cancer types.

### 3.4. The Associations of RELL2 with Immune Cell Infiltrating Levels and the Tumor Microenvironment

**Figure 7:**
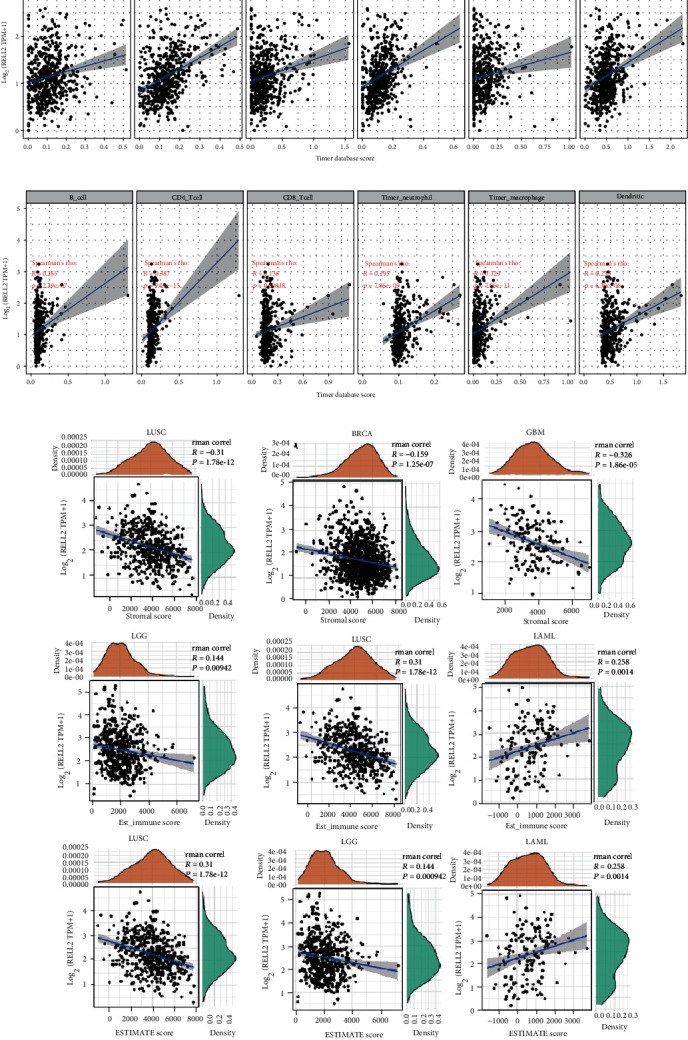
The relationships between RELL2 expression level and tumor immune infiltration in the TCGA database. (a) RELL2 expression and its correlations with the immune cell infiltration in KIRC. (b) Association of RELL2 expression with immune cell infiltration in LIHC. (c) Association of RELL2 expression levels and immune score, ESTIMATE immune score, and stromal score in pan-cancer analysis.

Tumor infiltrating lymphocytes (TILs) substantially contribute to and affect the OS of cancer patients and their lymph node status. We evaluated the associations of RELL2 with six distinct types of immune cells that influence the tumor microenvironment (TME). Our findings demonstrated that RELL2 was correlated with immune cells in different cancer types, particularly in KIRC and LIHC. In KIRC, a positive and significant correlation of RELL2 with four immune cell types was found, specifically CD4^+^ T cell, DCs, macrophages, and neutrophils (Figure 7(a)). In LIHC, RELL2 was positively and significantly correlated with five immune cell types: B cells, CD4^+^ T cells, DCs, macrophages, and neutrophils (Figure 7(b)).

To explore the associations of RELL2 with the TME, which contains immune, stromal, and tumor cells, we used the R package of ESTIMATE to determine the immune and stromal scores of every cancer type.  Figure 7(c) illustrates the top three cancers significantly correlated with RELL2 expression. Among the 33 cancers, the immune scores were BRCA, GBM, and LUSC. Significant correlations of the leading three tumors with the stromal score and RELL2 expression were LAML, LGG, and LUSC. The three leading cancer types that were significantly correlated with the RELL2 expression and immune score deduced using ESTIMATE were LAML, LGG, and LUSC. Overall, these outcomes underscored the significant and positive correlation of RELL2 expression levels with LAML in the stromal score and immune score inferred by ESTIMATE, as well as its negative correlation with LUSC, BRCA, and GBM in the immune score, and LGG and LUSC in the stromal score and immune score obtained using ESTIMATE.

### 3.5. Correlations between RELL2 Expression Levels and Immune Neoantigens and ICGs

**Figure 8:**
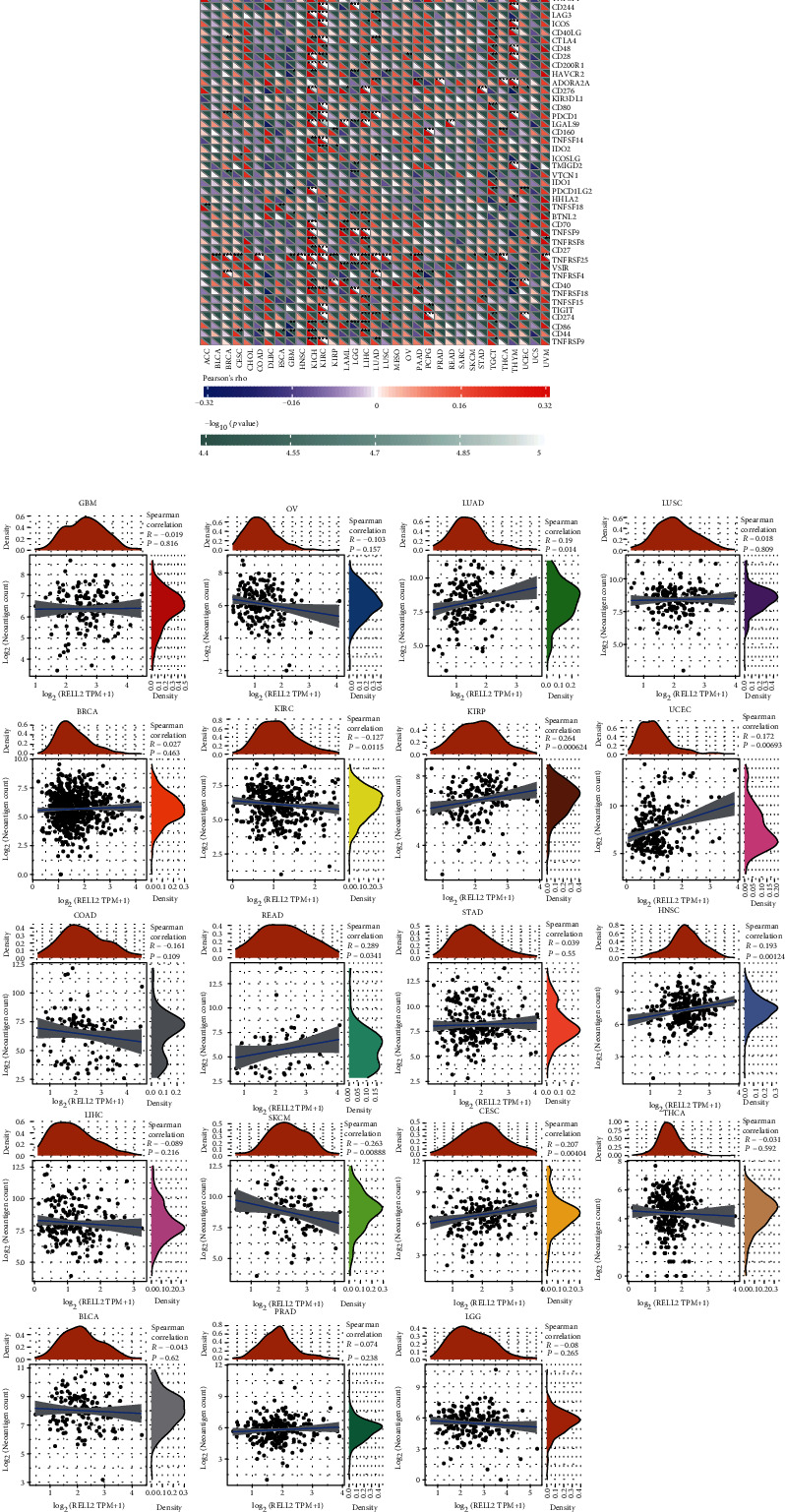
RELL2 expression level in ICG expression and neoantigens in different cancers. (a) Relation between RELL2 expression levels and key ICG expression. Statistical significance: ^∗^*P* < 0.05, ^∗∗^*P* < 0.01, and ^∗∗∗^*P* < 0.001. (b) RELL2 expression and its correlations with immune neoantigens across 19 types of tumors.

Recently, tumor immunotherapy has garnered considerable attention. We evaluated the correlation of RELL2 expression with over 40 standard checkpoint genes (Figure 8(a)). The findings demonstrated the significant correlation of RELL2 expression level with the expression of several checkpoint genes in different cancer types, particularly KICH, KIRC, and THYM. These results suggested the potential role of RELL2 in influencing tumor immunity through the regulation of these checkpoint genes.

We also analyzed the association of RELL2 expression levels with immune neoantigens in different tumors. The outcomes revealed a positive correlation of RELL2 expression level with three distinct immune antigens, specifically CESC, KIRP, and SKCM (Figure 8(b)).

### 3.6. The Correlations between RELL2 Expression and DNA MMR Genes and Methyltransferase Expression

**Figure 9:**
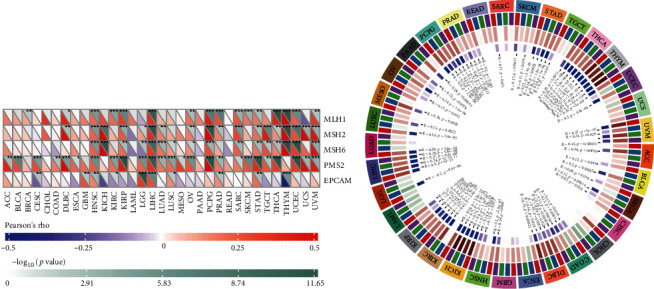
RELL2 expression level in 5 MMRs and 4 methyltransferases. (a) Relation between RELL2 expression and five MMR gene expressions based on pan-cancer analysis. (b) Relationships between RELL2 expression and four methyltransferases. DNMT1 is indicated in red, DNMT2 is indicated in blue, DNMT3a is indicated in green, and DNMT3b is indicated in purple.

MMRs can cause gene mutations to occur at a faster rate. We examined the relationship of RELL2 expression with numerous essential MMR genes and identified positive correlations of RELL2 expression with MutL homolog 1 (MLH1), MutS homolog 2 (MSH2), and MutS homolog 6 (MSH6) in UVM, UCEC, THYM, TGCT, STAD, SARC, PRAD, PCPG, OV, LIHC, KIRC, KICH, and HNSC. Conversely, RELL2 expression was negatively correlated with epithelial cell adhesion molecule (EpCAM) in THYM, TGCT, MESO, LAML, KIRP, KIRC, KICH, ESCA, and CESC (Figure 9(a)). We studied the correlation of RELL2 expression with four methyltransferases, DNMT3B, DNMT3A, DNMT2, and DNMT1. The results identified a positive correlation of RELL2 expression with methyltransferases in all tumors except for UCEC, UCS, CHOL, COAD, ESCA, LAML, and LGG (Figure 9(b)).

### 3.7. GSEA

**Figure 10:**
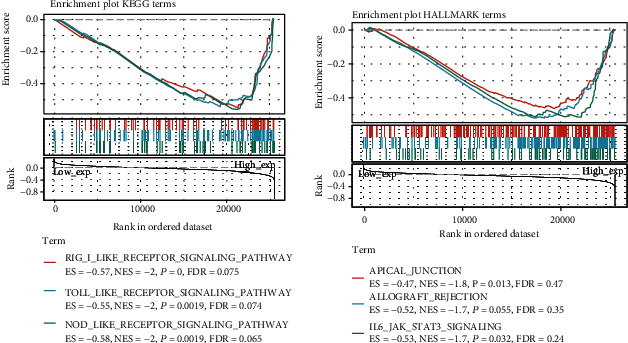
GSEA of RELL2 expression. (a) Gene set enrichment analysis results showing the top three signaling pathways correlated with RELL2 expression in the KEGG database. (b) Gene set enrichment analysis results showing the top three signaling pathways correlated with RELL2 expression in the hallmark database.

We classified all pan-cancer samples into the low- and high-expression groups using the median expression level of RELL2. We used GSEA to assess the enrichment of signaling pathways in KEGG and hallmark (false discovery rate < 0.25, *P* value < 0.05, ∣normalized enrichment score | >1) (Table 1).  Figure 10 lists the top three pathways that were considerably enriched in both databases; the most enriched signaling pathways were the RIG-I-like receptor (RLR) and apical junction pathways.

## 4. Discussion

Apoptosis, known as programmed cell death, is categorized into late apoptosis and early apoptosis and depends on the balance between apoptosis-promoting and apoptosis-inhibiting proteins [32, 33]. During apoptosis, n-tetradecane acylation can occur after caspase cleavage, exposing a hidden N-terminal glycine residue. The enzyme that is responsible for myristoylation is N-myristoyltransferase (NMT). In *Saccharomyces cerevisiae*, the loss of NMT function is fatal, and *Drosophila* lacking NMT have a variety of developmental defects [34]. In multicellular organisms, apoptosis is a highly regulated form of cell death. Mitochondria are the regulatory center of apoptosis and the site of the well-known intrinsic apoptosis pathway [35]. Apoptosis is regulated by external forces, such as death receptor signaling, and also intracellular milieu [36]. Apoptosis-inducing factor is a novel mediator in apoptosis and the most frequently studied tracer is Annexin-V [37]. Studies showed that orlistat induces apoptosis and bortezomib plays a significant role in sensitizing RPMI-8226 cells to apoptosis [38]. Other drugs interfering with apoptosis can also play an oncogenic role [39]. Furthermore, apoptosis-related genetic mutations or abnormal expression of apoptosis-related proteins may inhibit apoptosis and trigger tumorigenesis. Tumor treatment is vital in the induction of the expression of apoptosis-related genes to expedite tumor cell apoptosis [40]. In addition, a close connection may exist between apoptosis and autophagy. The process of apoptosis may start with autophagy and the process of autophagy may finish with apoptosis [41]. Like cell apoptosis and cell senescence, autophagy is a very important biological phenomenon that participates in the development and growth of organisms and other processes. Autophagy plays a great role in the metabolic balance of the human body, but excessive autophagy can cause damage. While enhancing autophagy in normal cells can inhibit tumorigenesis, tumor cells also counter therapeutic drug-, metabolites-, and hypoxia-induced stress response. Excessive autophagy may engulf normal cells, leading to impaired body function.

In our study, we found that RELL2 is overexpressed in most cancers. A previous study revealed that RELL2 overexpression is correlated with apoptosis [1]. We investigated the expression level of RELL2 through several databases such as TCGA, CCLE, GTEx, and TIMER and found that RELL2 is overexpressed in many tumor tissues. We further explored the prognostic value of RELL2 by analyzing the association of RELL2 expression with OS, DSS, DFI, and PFI in various cancer types. The results showed RELL2 plays an independent role in many cancers. Subsequent survival analysis revealed that the overexpression of RELL2 predicted a poor survival outcome of cancer patients. Next, we explored the correlations between RELL2 expression level and different stages, TMB, and MSI. We found that RELL2 expression correlated with increased stages in many cancers, suggesting that high expression level of RELL2 may predict the degree of malignancy of the tumor. Increasing studies have revealed that the tumor microenvironment plays a vital role in tumor progression. We then explored the relationships between RELL2 and different immune checkpoints and found that RELL2 was significantly expressed in many cancers. We analyzed the correlations between RELL2 and immune cells, MMR genes, and DNA methylation. The results showed that RELL2 was positively and significantly expressed in many immune cells and tumors.

In our gene enrichment analysis, we found that the expression of RELL2 was associated with a number of metabolic pathways and metabolism, including the Toll-like receptor signaling pathway, Rig I-like receptor signaling pathway, chemokine signaling pathway, T cell receptor signaling pathway, JAK-STAT signaling pathway, B cell receptor signaling pathway, glycerolipid metabolism, galactose metabolism, and IL6-JAK-STAT3 signaling. These findings highlight the significant correlation of RELL2 with the control of signaling pathways and its substantial influence in regulating the tumor microenvironment and body metabolism. In HCC, tumors gain immune escape, which speeds up the occurrence and development of cancer through apoptosis [42]. Several other papers showed the ability of tumor cells to resist immune cell–triggered apoptosis, enabling escape from the host's immune monitoring [43]. Our results identified a correlation of RELL2 with MDSCs, CD4+ T cells, and CD8+ T cells and the correlation of RELL2 with tumor immune infiltration. Ultimately, these findings suggests that these signaling pathways across various cancer types enable the escape from tumors through apoptosis. Thus, blocking the apoptosis pathway may be an efficient method to improve tumor-targeted therapy. Studies showed that inhibition of apoptosis can improve the survival of muscle cells [44]. However, apoptosis is complex and its targets are various. Therefore, identifying an optimal apoptosis-targeting gene seems to be the main approach to improve tumor-targeted therapy inhibiting apoptosis.

RELL2 overexpression is associated with apoptosis [1]. We found that RELL2 expression was significantly correlated with the majority cancers through our pan-cancer analysis and was highly expressed in tumor cells. High expression of RELL2 correlates with a poor prognostic outcome in most cancers. Thus, RELL2 may influence the survival outcome through apoptosis. We also found that RELL2 was correlated with the tumor microenvironment and immune cells in many cancers, indicating that RELL2 in the tumor microenvironment and immune cells may regulate the occurrence and development of tumors. Together, these findings indicate that RELL2 may stimulate tumor progression in many cancer types and lead to an unsatisfactory survival outcome.

Our study has several limitations. Our findings are derived from bioinformatics analyses and experimental data are lacking. Further studies are warranted to validate our results. A large sample size and comprehensive analysis of RELL2 can provide insight for future exploration of the role of RELL2 in cancer.

## Figures and Tables

**Table 1:** The GSEA enrichment results of KEGG and HALLMARK terms (∣NES | >1, *P* value < 0.05, FDR < 0.25).

Term	ES	NES	NP	FDR	FWER
KEGG_RIG_I_LIKE_RECEPTOR_SIGNALING_PATHWAY	-0.5688	-2.0268	0	0.0746	0.036
KEGG_TOLL_LIKE_RECEPTOR_SIGNALING_PATHWAY	-0.5527	-1.9775	0.0019	0.0736	0.066
KEGG_NOD_LIKE_RECEPTOR_SIGNALING_PATHWAY	-0.5794	-1.9616	0.0019	0.0654	0.085
KEGG_CHEMOKINE_SIGNALING_PATHWAY	-0.5058	-1.8962	0.0019	0.1004	0.146
KEGG_FC_EPSILON_RI_SIGNALING_PATHWAY	-0.505	-1.8811	0.0039	0.0971	0.163
KEGG_BLADDER_CANCER	-0.5343	-1.8675	0	0.0908	0.184
KEGG_T_CELL_RECEPTOR_SIGNALING_PATHWAY	-0.5332	-1.8303	0.004	0.114	0.244
KEGG_FC_GAMMA_R_MEDIATED_PHAGOCYTOSIS	-0.5035	-1.8171	0.0097	0.1117	0.268
KEGG_ACUTE_MYELOID_LEUKEMIA	-0.5195	-1.7903	0.0075	0.1331	0.323
KEGG_PRIMARY_IMMUNODEFICIENCY	-0.6838	-1.7854	0.0243	0.1243	0.333
KEGG_EPITHELIAL_CELL_SIGNALING_IN_HELICOBACTER_PYLORI_INFECTION	-0.4955	-1.7849	0.0096	0.1136	0.335
KEGG_PHOSPHATIDYLINOSITOL_SIGNALING_SYSTEM	-0.4897	-1.7749	0.0096	0.1163	0.359
KEGG_NATURAL_KILLER_CELL_MEDIATED_CYTOTOXICITY	-0.4815	-1.7648	0.0076	0.1188	0.378
KEGG_JAK_STAT_SIGNALING_PATHWAY	-0.4522	-1.7425	0.004	0.1311	0.421
KEGG_B_CELL_RECEPTOR_SIGNALING_PATHWAY	-0.5203	-1.7374	0.0314	0.1278	0.434
KEGG_CYTOSOLIC_DNA_SENSING_PATHWAY	-0.4982	-1.726	0.0134	0.1318	0.461
KEGG_VEGF_SIGNALING_PATHWAY	-0.4338	-1.7078	0.0058	0.1449	0.51
KEGG_SNARE_INTERACTIONS_IN_VESICULAR_TRANSPORT	-0.5141	-1.7037	0.0155	0.141	0.514
KEGG_CYTOKINE_CYTOKINE_RECEPTOR_INTERACTION	-0.4357	-1.681	0.025	0.1564	0.555
KEGG_NON_SMALL_CELL_LUNG_CANCER	-0.482	-1.6683	0.018	0.1627	0.584
KEGG_PROGESTERONE_MEDIATED_OOCYTE_MATURATION	-0.4712	-1.6661	0.0198	0.1577	0.585
KEGG_ENDOCYTOSIS	-0.4431	-1.6584	0.0238	0.1586	0.598
KEGG_GLYCEROPHOSPHOLIPID_METABOLISM	-0.4131	-1.6561	0.0153	0.1535	0.603
KEGG_GLYCEROLIPID_METABOLISM	-0.448	-1.6531	0.0188	0.1508	0.616
KEGG_NEUROTROPHIN_SIGNALING_PATHWAY	-0.4384	-1.6308	0.0234	0.1666	0.66
KEGG_HOMOLOGOUS_RECOMBINATION	-0.6222	-1.6064	0.043	0.176	0.706
KEGG_APOPTOSIS	-0.4399	-1.6034	0.0356	0.1725	0.707
KEGG_SMALL_CELL_LUNG_CANCER	-0.4467	-1.5996	0.0334	0.1708	0.711
KEGG_FRUCTOSE_AND_MANNOSE_METABOLISM	-0.4904	-1.5882	0.0262	0.1667	0.733
KEGG_MAPK_SIGNALING_PATHWAY	-0.3707	-1.571	0.0275	0.1767	0.754
KEGG_NOTCH_SIGNALING_PATHWAY	-0.4842	-1.5703	0.0414	0.1724	0.755
KEGG_VIBRIO_CHOLERAE_INFECTION	-0.4357	-1.5702	0.0457	0.1678	0.756
KEGG_GNRH_SIGNALING_PATHWAY	-0.3912	-1.5666	0.0251	0.1674	0.766
KEGG_GLIOMA	-0.4327	-1.5505	0.0378	0.17	0.792
KEGG_AMINO_SUGAR_AND_NUCLEOTIDE_SUGAR_METABOLISM	-0.4738	-1.5488	0.049	0.1673	0.793
KEGG_GALACTOSE_METABOLISM	-0.5049	-1.5187	0.0432	0.1853	0.837
KEGG_ABC_TRANSPORTERS	-0.4218	-1.5138	0.0329	0.1834	0.847
KEGG_CALCIUM_SIGNALING_PATHWAY	-0.3402	-1.4528	0.0345	0.2133	0.913
KEGG_NEUROACTIVE_LIGAND_RECEPTOR_INTERACTION	-0.3304	-1.4135	0.034	0.2292	0.938
KEGG_RIBOSOME	0.8522	1.8217	0.0078	0.2079	0.27
HALLMARK_IL6_JAK_STAT3_SIGNALING	-0.5296	-1.7056	0.032	0.2416	0.236
HALLMARK_INFLAMMATORY_RESPONSE	-0.4701	-1.6624	0.0415	0.1665	0.283
HALLMARK_IL2_STAT5_SIGNALING	-0.3901	-1.5822	0.0394	0.2345	0.398

## Data Availability

The data used in our study came from public databases and are freely available at the following sites: TCGA: https://portal.gdc.cancer.gov/; CCLE: https://sites.broadinstitute.org/ccle; GTEx: https://gtexportal.org/; and TIMER: https://cistrome.shinyapps.io/timer/.
